# Paternity leave, mental health and wellbeing for new parents: evidence from a national survey in the UK

**DOI:** 10.1016/j.ssmph.2025.101811

**Published:** 2025-05-07

**Authors:** Emily Humphreys, Stephen O'Neill, Veronique Filippi, Emilie Courtin

**Affiliations:** a London School of Hygiene & Tropical Medicine, 15–17 Tavistock Place, London, WC1H 9SH, UK; b London School of Economics, Houghton Street, London, WC2A 2AE, UK

## Abstract

Paternity leave has the potential to help parents by enabling new fathers to spend time with their families. However, existing evidence about its association with parental mental health and wellbeing is mixed.

This study used data from Understanding Society, a national UK household survey, to examine uptake of paternity leave and its association with measures of mental health and wellbeing for fathers (n = 1385) and mothers (n = 1384) of infants born 2009–2019. We used logistic regression to explore paternity leave uptake and inverse probability weighted regression adjustment (IPWRA) to estimate the association between paternity leave uptake and the mental wellbeing (Short Form-12 Mental Component Score (SF-12 MCS)) and mental health (General Health Questionnaire-12 (GHQ-12) caseness) of fathers and mothers in the months after the birth of their child.

Odds of taking paternity leave were higher for more educated fathers and those born in the UK. After adjusting for potential confounders, we found no strong evidence of association between paternity leave and mental wellbeing or mental health of mothers or fathers in our overall sample. This finding was robust to a range of sensitivity analyses including alternative model specifications, imputation of missing data, and weighting. However, subgroup analysis showed that fathers with above median household incomes had better mental wellbeing if they took paternity leave (1.43-point difference in SF-12 MCS; 95 % CI 0.25,2.62; p = 0.02).

Improved policies are needed to ensure parental leave reduces inequalities in mental health and wellbeing.

## Highlights

• Paternity leave is associated with better mental wellbeing for high-income fathers.
• Little evidence of association with mental health or wellbeing of mothers or low-income fathers.
• Results are robust to a range of sensitivity analyses.
• Policymakers should consider potential equity effects of parental leave policies.

## 1 Introduction

### 1.1 Background and theoretical rationale

The peri- and post-natal periods are seen as sensitive for the future health of parents, who experience neurobiological changes; sleep disturbance; and new demands on their time, money, and emotional resources (Saxbe et al., 2018). Postpartum depression is common in mothers (Shorey et al., 2018) and fathers (Rao et al., 2020).

To support parents, most countries have introduced statutory entitlements to paid maternity, paternity or parental leave, a period of employment protection allowing new parents to take time away from work (Organization for Economic Cooperation and Development, 2024). Paid maternity leave is available in 96 % of countries and paid paternity leave in 56 % of countries (Earle et al., 2023).

Paid paternity leave might affect mental health for several reasons. The conceptual framework underpinning this study (appendix A) suggests that leave policy and access rights affect parents’ choices and experiences. These include whether they take leave from work; its duration; their income; and whether they take leave simultaneously with another parent.

Paternity leave experiences may influence each parent's role in childcare and their relationships with each other and with their child, which could promote good mental health. If fathers take paternity leave, mothers may benefit from improved partner support, which is protective against postnatal depression (Yim et al., 2015). Both parents could see improved wellbeing through giving fathers more opportunities to participate in caregiving (Heymann et al., 2017). Research has found paternity leave to be associated with parental relationship stability (Petts, Carlson, & Knoester, 2020), and better child perceptions of father-child relationships (Petts, Knoester, & Waldfogel, 2020).

Adequately paid paternity leave of sufficient duration could also affect a family's ability to support their health by giving parents time for effective parenting and resources to maintain a healthy lifestyle. For example, there is evidence that paternity leave is associated with breastfeeding of infants among mothers (Flacking et al., 2010) and with increased physical activity among fathers (Johansson et al., 2014). Overall good family health could enhance parental mental health and wellbeing.

Effects of taking paid paternity leave might differ between groups. Paternity leave could particularly help first-time parents, who can feel unprepared (Refaeli et al., 2024); parents with low socioeconomic status or experiencing financial strain, who are at higher risk of parental postpartum depression (Ansari et al., 2021; O'Hara & McCabe, 2013; Wang et al., 2021); or first-generation migrants, who are at high risk of mental health problems (Close et al., 2016).

There is limited quantitative evidence about paternity leave and mental health and wellbeing for either mothers or fathers, summarised in Appendix B. Different outcomes, settings, and measures of exposure to paternity leave have been examined and findings are mixed. Studies have found paternity leave to be associated with better (Bilgrami et al., 2020; Cardenas et al., 2021; Honkaniemi et al., 2021; Lidbeck et al., 2018; Perry-Jenkins et al., 2017; Philpott & Corcoran, 2018; Redshaw & Henderson, 2013; Seimyr et al., 2004), worse (Barry et al., 2023; Månsdotter & Lundin, 2010; Séjourné et al., 2012), or similar (Feldman et al., 2004; Nishigori et al., 2020) mental health or wellbeing for one or both parents.

We hypothesised that paternity leave would improve mental health and wellbeing for fathers and mothers. We also hypothesised that effects would be greater for first-time parents, lower-income families and first-generation migrants.

### 1.2 Setting

The UK is an interesting setting for parental leave research. It has historically been aligned with a ‘male breadwinner’ model (Ciccia & Verloo, 2012). The proportion of women's time dedicated to childcare is among the highest in the OECD (Organization for Economic Co-operation and Development, 2010). It was the last EU member state to adopt a parental leave directive in 1999 (HAAS, 2003), and has a market-oriented care model and a wide gender gap in leave uptake intentions (Olsson et al., 2023). Employers implement their own parental leave policies above statutory minimums and they make a range of different provisions (Koslowski & Kadar-Satat, 2019), with resultant inequalities. The UK particularly contrasts with Scandinavian countries, which have high levels of gender equality and investment in social welfare (Andersen et al., 2017).

As of early 2025, paternity leave is not a universal entitlement in the UK, but requires a period of continuous employment.^1^ With its two weeks duration, the statutory paid leave earmarked for fathers is among the shortest in the OECD, where the average is 10.2 weeks (Organization for Economic Cooperation and Development, 2024). The statutory paternity payment rate in the UK midway through the time of this study in 2014 was 20 % of national average earnings; this compares to a rate of 76 % in Sweden (Organization for Economic Cooperation and Development, 2024), where much of the existing research evidence about paternity leave and mental health has been carried out.

Experiences of paternity leave in the UK may differ from those in countries with generous family policy entitlements. However, there is limited peer-reviewed UK evidence about association between paternity leave and parental mental health. Qualitative research has found working fathers expressing feelings of role strain and guilt (Darwin et al., 2017). A quantitative study using cross-sectional data from 2010 found lower self-reported depression at three months among mothers whose partner had taken paternity leave (Redshaw & Henderson, 2013), but it did not use a validated measure of mental wellbeing and did not examine outcomes among fathers.

To address this gap, this paper uses a large national survey dataset to contribute evidence about the association between paternity leave, mental wellbeing and mental health for mothers and fathers in the UK. It also reports on the characteristics associated with uptake of paternity leave and on differences in outcomes between subgroups.

## 2 Methods

### 2.1 Data and measures

We used data from Understanding Society (Institute for Social and Economic Research, 2022a). This is an ongoing survey beginning in 2009 with a nationally representative sample of approximately 26,000 households (Institute for Social and Economic Research, 2022b). It collects data on health, work, education, income, family and social life in the UK. Household and individual questionnaires are used for consenting participants aged ≥16. Respondents are re-interviewed approximately once per year.

To maximise sample size, we pooled data about new parents with babies born between the initiation of the survey and 2019, using waves 1–10 (2009–2020). For each parent, we used data from two survey waves. Baseline covariate data came from the wave before their child was born. Exposure and outcome data were from the wave after their child was born, when any short-term effects of paternity leave might be clearest. The exposure was a binary variable capturing whether a father had taken paternity leave; the outcomes were validated measures of mental health and wellbeing detailed below.

Survey participants were eligible for inclusion if (1) they were the biological mother or father of a newborn survey entrant when interviewed in any of waves 2–10 (2010–2020) (2) the parents lived together in the survey waves before and after the birth (3) fathers were continuously employed by the same employer over the same two waves (a proxy for paternity leave eligibility) and (4) complete data were available on their exposure and outcome (see appendix C). Parents appear once in the sample for each birth date, meaning that there is one record if they had twins or triplets, but separate records if they had children born at different times.

In the included sample of fathers (n = 1385), those who said they had taken or were currently taking paternity leave were defined as the exposed group; others were unexposed. Mothers (n = 1384) were defined as exposed if the father of their child had taken or was taking paternity leave.^2^ During the time covered by this study, fathers could only report one period of paternity leave in each wave of Understanding Society, so those who had more than one child between waves (n < 5) were assumed to have been consistent in their uptake of paternity leave in the main analysis and excluded in a sensitivity analysis.

The primary outcome measure was the Mental Component Score (MCS) of the SF-12. This is a generic self-completed wellbeing questionnaire with twelve questions covering eight domains of health and functioning (McDowell, 2006; Ware et al., 1996). Both the MCS and the Physical Component Score (PCS) have been shown to replicate results from the longer SF-36 in a UK population (Jenkinson & Layte, 1997). The MCS combines responses into a mental functioning score ranging from 0 to 100, transformed to have a mean of 50 and standard deviation of 10 in a reference population (Ware et al., 1995). The secondary outcome is ‘caseness’ – screening positive - on the 12-point General Health Questionnaire (GHQ-12). This indicates a probable mental health condition based on a score of ≥4 on a scale ranging from 0 to 12. It measures symptoms of mental distress and screens for current mental health conditions, but does not distinguish between them (McDowell, 2006).

Additional data on characteristics of each parent included their age when their child was born; the age of their child at the time of interview; the season of the birth (spring, summer, autumn, winter); whether they already had children; their ethnic group; the age of the youngest child in their household before the birth; their highest qualification; their monthly net household income adjusted to 2015 prices using the consumer price index; their country of birth (UK or elsewhere); the National Statistics-Socio Economic Classification (NS-SEC) of their job; the current parental leave policy regime at the time of the birth; and their SF-12 MCS and PCS before the birth. The mother's pre-birth employment status was also included.

### 2.2 Study design and analysis

This is an analytical, rather than descriptive, study based on survey data from new parents. Although the sample is cross-sectional, the study is strengthened by the inclusion of covariate data which was collected before the birth. We stratified the data to report separately about mothers and fathers.

We used three logistic regression models to identify which parental characteristics were independently associated with taking paternity leave in the father sample. Model (1) adjusted for demographic characteristics of both parents; model (2) added socioeconomic characteristics; and the fully adjusted model (3) also included parents’ pre-birth mental and physical wellbeing measured by the SF-12 MCS and PCS.

To identify association between paternity leave and mental health and wellbeing, we used the same covariates in inverse probability weighted regression adjustment (IPWRA) models, using paternity leave as the exposure and post-birth mental health and wellbeing measures as outcomes (see Appendix D for further methodological details). Our fully adjusted IPWRA models used a linear outcome model for the continuous primary outcome (SF-12 MCS) and a logit outcome model for the binary secondary outcome (GHQ-12 caseness). Standard errors were clustered at the individual and PSU level to take account of the survey design and the possibility of autocorrelation of outcomes for people who appeared in the sample more than once. We report results as the point difference in mean score for the continuous primary outcome (SF-12 MCS) and risk difference for the binary secondary outcome (GHQ-12 caseness).

Four subgroup analyses were carried out. We considered (1) parents above and below the median net household monthly income, (2) parents who were and were not born in the UK and (3) first time parents and those who already had children. In each case, these were carried out by stratifying both the mother and father datasets by the subgroup identifier and conducting the analysis separately for each stratum. Finally (4), for fathers with known paternity leave duration, we created a categorical exposure variable identifying leave length of ≤2 weeks or >2 weeks and compared each with those who took no paternity leave.

We ran four sets of sensitivity analyses to assess the robustness of the results. First, we applied different choices of analytical model. In addition to the IPWRA models, we used models based on regression adjustment alone; inverse probability weighting alone; and propensity score matching.

Second, we examined the sensitivity of our findings to using weighted or unweighted data. This is because there is some controversy over whether to use weights in analytic models based on complex survey data (Bollen et al., 2016; West et al., 2018). The weighted dataset consists of people living in a representative sample of UK households at the beginning of the survey and adjusts for non-response; the unweighted dataset used for the main analysis also includes any partners who joined their households in a subsequent wave. It does not constitute a representative sample of the population.

Third, we assessed the impact of missing data. The primary analysis used complete cases. However, if data for incomplete cases was not missing completely at random, this would lead to biased results. Sensitivity analysis was carried out for the primary outcome analysis using imputed covariate data. Multiple imputation with chained equations was used to impute a number of datasets (n = 15) selected to exceed the percentage of cases with any missing data, as recommended by White and colleagues (2011). Imputation models included all analytical model variables. Values of missing categorical variables were imputed using a multinomial logit model and values of continuous variables were imputed using predictive mean matching to ensure the imputed values remained within a feasible range (Harel et al., 2018). Analysis results from all imputed datasets were combined under Rubin's rules (Rubin, 1987).

Finally, we tested the impact of using a lower ‘caseness’ threshold of ≥3 for the GHQ-12 secondary outcome, since the optimal threshold is unclear (McDowell, 2006). This lower threshold would have higher sensitivity to detect possible cases of mental ill-health.

Data preparation and analysis used Stata 18.

## 3 Results

### 3.1 Sample

Of 2647 respondent families, approximately a third (34 %) were excluded because the fathers were not continuously employed by the same employer and were therefore unlikely to be eligible for statutory paternity leave. Of the remaining 1741 families, complete data were available for samples of 1385 fathers and 1384 mothers.^2^ An additional 216 fathers and 217 mothers with complete information on outcome and exposure but missing covariate data were excluded from the main analysis but included in the sensitivity analysis using imputed data, giving samples of 1601 fathers and 1601 mothers (see Appendix C).

In total, 1084 fathers (78 %) reported taking some paternity leave. Fathers were aged 35 years on average; mothers 32 years. At the time of interview, the average age of the children was 6.5 months in the sample of fathers and 6.4 months in the sample of mothers. This differed between those taking and not taking paternity leave. Most families (65 %) already had at least one child. The mean monthly household income was £3632 (2015 prices); higher than the national average. There were differences in uptake of paternity leave by mother's age, child's age, season of birth, ages of pre-existing children, maternal employment status and household income, as well as ethnic group, qualifications, socioeconomic categories, places of birth and prior physical health of both parents (see Table 1).

**Table 1:** Descriptive statistics.

	Father sample (n = 1385)			Mother sample (n = 1384)		
	Paternity leave taken			Paternity leave taken		
	No N (%/sd)	Yes N (%/sd)	p	No N (%/sd)	Yes N(%/sd)	p
N	301 (12 %)	1084 (78 %)		305 (22 %)	1079 (78 %)	
Father's age at birth, years (mean)	35 (6.1)	35 (5.6)	0.79	35 (6.1)	35 (5.6)	0.85
Mother's age at birth, years (mean)	31 (4.6)	32 (4.6)	<0.01	32 (4.6)	32 (4.7)	0.01
Time since birth, months	7.4 (4.8)	6.2 (3.8)	<0.01	7 (4.8)	6 (3.8)	<0.01
Child's season of birth						
Winter	62 (21 %)	260 (24 %)	0.12	64 (21 %)	256 (24 %)	0.12
Spring	69 (23 %)	295 (27 %)		68 (22 %)	294 (27 %)	
Summer	85 (28 %)	254 (23 %)		85 (28 %)	256 (24 %)	
Autumn	85 (28 %)	275 (25 %)		88 (29 %)	273 (25 %)	
Pre-existing children						
Neither parent already has children	95 (32 %)	412 (38 %)	0.12	96 (31 %)	411 (38 %)	0.10
One parent already has≥1 child	14 (5 %)	48 (4 %)		14 (5 %)	48 (4 %)	
Both parents already have≥1 child	192 (64 %)	624 (58 %)		195 (64 %)	620 (57 %)	
Age of youngest child in household before the birth (years)	2 (2.6)	1.7 (2.3)	0.02	2 (2.6)	2 (2.3)	0.02
Father's ethnic group						
White British	224 (74 %)	891 (82 %)	<0.01	226 (74 %)	888 (82 %)	<0.01
Asian or Asian British	45 (15 %)	96 (9 %)		46 (15 %)	95 (9 %)	
Black, Black British, mixed, other white, mixed or other ethnic group	32 (11 %)	97 (9 %)		33 (11 %)	96 (9 %)	
Mother's ethnic group						
White British	216 (74 %)	877 (81 %)	<0.01	219 (72 %)	874 (81 %)	<0.01
Asian or Asian British	46 (15 %)	102 (9 %)		47 (15 %)	101 (9 %)	
Black, Black British, mixed, other white, mixed or other ethnic group	39 (13 %)	105 (10 %)		39 (13 %)	104 (10 %)	
Mother's pre-birth employment status						
Employed or self-employed	199 (66 %)	858 (79 %)	<0.01	205 (67 %)	859 (80 %)	<0.01
Maternity leave, family care, shared parental leave, or other	102 (34 %)	226 (21 %)		100 (33 %)	220 (20 %)	
Monthly household income before birth, £1,000s, 2015 prices (mean)	3.40 (1.6)	3.70 (1.9)	<0.01	3.40 (1.6)	3.70 (1.7)	0.01
Father's highest qualification						
Degree or other higher	135 (45 %)	613 (57 %)	<0.01	136 (45 %)	607 (56 %)	<0.01
A-level etc (typically awarded aged 18)	72 (24 %)	274 (25 %)		75 (25 %)	271 (25 %)	
GCSE etc (typically awarded aged 16)	68 (23 %)	167 (15 %)		68 (22 %)	170 (16 %)	
Other or no qualification	26 (9 %)	30 (3 %)		26 (9 %)	31 (3 %)	
Mother's highest qualification						
Degree or other higher	173 (57 %)	714 (66 %)	0.04	177 (58 %)	710 (66 %)	0.08
A-level etc (typically awarded aged 18)	61 (20 %)	190 (18 %)		64 (21 %)	193 (18 %)	
GCSE etc (typically awarded aged 16)	51 (17 %)	143 (13 %)		49 (16 %)	140 (13 %)	
Other or no qualification	16 (5 %)	37 (3 %)		15 (5 %)	36 (3 %)	
NS-SEC of father's job before the birth						
Management & professional	147 (49 %)	621(57 %)	<0.01	152 (50 %)	616 (57 %)	<0.01
Intermediate, small employers & own account	33 (11 %)	149 (14 %)		33 (11 %)	152 (14 %)	
Lower supervisory & technical	38 (13 %)	120 (11 %)		39 (13 %)	119 (11 %)	
Semi-routine & routine	83 (28 %)	194 (18 %)		81 (27 %)	192 (18 %)	
NS-SEC of mother's job before the birth						
Management & professional	119 (40 %)	548 (51 %)	<0.01	126 (41 %)	547 (51 %)	<0.01
Intermediate, small employers & own account	38 (13 %)	188 (17 %)		38 (12 %)	188 (17 %)	
Lower supervisory & technical	11 (4 %)	31 (3 %)		11 (4 %)	32 (3)	
Semi-routine & routine	52 (17 %)	134 (12 %)		52 (17 %)	136 (13 %)	
Inapplicable	81 (27 %)	183 (17 %)		78 (26 %)	176 (16 %)	
Policy regime						
Pre-Additional Paternity Leave (before April 2011)	47 (16 %)	199 (18 %)	0.30	48 (16 %)	199 (18 %)	0.29
Additional Paternity Leave (April 2011–March2015)	157 (52 %)	513 (47 %)		160 (52 %)	513 (48 %)	
Shared Parental Leave (since April 2015)	97 (32 %)	372 (34 %)		97 (32 %)	367 (34 %)	
Parents' place of birth						
Both parents born in the UK	210 (70 %)	872 (80 %)	<0.01	214 (70 %)	867 (80 %)	<0.01
Mother born outside the UK	26 (9 %)	87 (8 %)		25 (8 %)	85 (8 %)	
Father born outside the UK	24 (8 %)	45 (4 %)		26 (9 %)	49 (5 %)	
Both parents born outside the UK	41 (14 %)	80 (7 %)		40 (13 %)	78 (7 %)	
Father's pre-birth SF-12 MCS (mean)	51 (7.6)	51 (7.6)	0.46	51 (7.8)	51 (7.6)	0.63
Father's pre-birth SF-12 PCS (mean)	54 (6.3)	55 (6.0)	0.04	54 (6.3)	55 (6.1)	0.05
Mother's pre-birth SF-12 MCS (mean)	50 (9.1)	49 (8.7)	0.63	50 (9.1)	50 (8.7)	0.49
Mother's pre-birth SF-12 PCS (mean)	51 (8.8)	52 (8.4)	0.02	51(8.7)	52 (8.4)	0.01

Reported p-values reflect unadjusted comparisons between those who took and did not take paternity leave, based on t-tests for a difference between means for continuous variables and chi-squared tests for categorical variables.

### 3.2 Paternity leave uptake

Results of logistic regression models for paternity leave uptake in the sample of fathers are presented in Table 2. The median duration of leave, calculated as the difference between start and end dates among those who specified this information (n = 1022), was two weeks.

**Table 2:** Results of logistic regression models to identify predictors of paternity leave uptake in father sample.

	Model 1 Demographic		Model 2 (1) + socioeconomic		Model 3 (2) + health/wellbeing	
	AOR	95 % CI	AOR	95 % CI	AOR	95 % CI
Father's age at birth	0.97	(0.94–1.00)	0.97	(0.94–1.00)	0.97	(0.94–1.00)
Mother's age at birth	1.07	(1.03–1.12)	1.06	(1.01–1.10)	1.06	(1.02–1.10)
Time since birth (months)	0.93	(0.90–0.96)	0.93	(0.89–0.96)	0.93	(0.89–0.96)
Pre-existing children						
Neither parent already has children	1		1		1	
One parent already has ≥1 child	0.74	(0.39–1.39)	0.93	(0.46–1.88)	0.92	(0.45–1.90)
Both parents already have ≥1 child	0.71	(0.53–0.95)	0.86	(0.60–1.24)	0.87	(0.60–1.25)
Child's season of birth						
Winter	1		1		1	
Spring	1.02	(0.69–1.51)	1.06	(0.71–1.58)	1.06	(0.70–1.58)
Summer	0.65	(0.45–0.95)	0.65	(0.44–0.97)	0.65	(0.44–0.97)
Autumn	0.77	(0.53–1.12)	0.77	(0.52–1.14)	0.77	(0.52–1.15)
Father's ethnic group						
White British	1		1		1	
Asian or Asian British	0.76	(0.38–1.51)	1.05	(0.46–2.40)	1.04	(0.45–2.38)
Black, Black British, mixed, other white, mixed or other ethnic group	0.94	(0.59–1.51)	1.57	(0.81–3.02)	1.59	(0.82–3.07)
Mother's ethnic group						
White British	1		1		1	
Asian or Asian British	0.7	(0.35–1.39)	0.8	(0.34–1.86)	0.8	(0.34–1.86)
Black, Black British, mixed, other white, mixed or other ethnic group	0.64	(0.41–1.02)	0.72	(0.39–1.31)	0.72	(0.39–1.32)
Monthly household income before birth, £1,000s (2015 prices)			1.03	(0.96–1.12)	1.04	(0.96–1.12)
Age of youngest child in household before the birth (years)			0.99	(0.92–1.06)	0.99	(0.92–1.06)
Mother's pre-birth employment status						
Employed or self-employed			1		1	
Maternity leave, family care, shared parental leave, or other			0.69	(0.42–1.15)	0.68	(0.41–1.15)
Father's highest qualification						
Degree or other higher			1		1	
A-level etc			0.9	(0.62–1.30)	0.89	(0.62–1.30)
GCSE etc			0.56	(0.36–0.86)	0.56	(0.36–0.87)
Other or no qualification			0.3	(0.16–0.56)	0.3	(0.16–0.56)
Mother's highest qualification						
Degree or other higher			1		1	
A-level etc (typically awarded at age 18)			1.05	(0.70–1.58)	1.05	(0.70–1.57)
GCSE etc (typically awarded at age 16)			1.07	(0.70–1.65)	1.08	(0.70–1.67)
Other or no qualification			1.25	(0.62–2.50)	1.23	(0.61–2.49)
NS-SEC of father's job before the birth						
Management & professional			1		1	
Intermediate, small employers & own account			1.18	(0.76–1.82)	1.17	(0.75–1.81)
Lower supervisory & technical			0.91	(0.57–1.44)	0.89	(0.56–1.43)
Semi-routine & routine			1.06	(0.71–1.58)	1.04	(0.69–1.55)
NS-SEC of mother's job before the birth						
Management & professional			1		1	
Intermediate, small employers & own account			1.27	(0.81–1.98)	1.26	(0.81–1.97)
Lower supervisory & technical			0.73	(0.34–1.55)	0.75	(0.35–1.59)
Semi-routine & routine			0.77	(0.50–1.19)	0.76	(0.49–1.19)
Inapplicable			1.09	(0.59–2.01)	1.1	(0.59–2.03)
Policy regime						
Pre-Additional Paternity Leave			1		1	
Additional Paternity Leave			0.69	(0.47–1.03)	0.68	(0.45–1.01)
Shared Parental Leave			0.76	(0.50–1.17)	0.74	(0.48–1.14)
Parents' place of birth						
Both parents born in the UK			1		1	
Mother born outside the UK			0.91	(0.49–1.69)	0.93	(0.50–1.71)
Father born outside the UK			0.48	(0.27–0.89)	0.49	(0.27–0.90)
Both parents born outside the UK			0.5	(0.25–0.99)	0.5	(0.25–0.99)
Mother's pre-birth SF-12 MCS					0.99	(0.98–1.01)
Mother's pre-birth SF-12 PCS					1	(0.98–1.01)
Father's pre-birth SF-12 MCS					0.99	(0.97–1.01)
Father's pre-birth SF-12 PCS					1	(0.98–1.02)
Intercept	3.02		6.84	(2.06–22.69)	14.91	(1.51–146.87)

Parental age was associated with reported paternity leave uptake in all three models. In the fully adjusted model, fathers were more likely to report taking paternity leave if the mother of the baby was older (adjusted odds ratio (AOR) 1.06 for each year's increase in age; 95 % CI 1.02,1.10; p < 0.01). Older fathers were less likely to report paternity leave (AOR 0.97 for each year's increase in age; 95 % CI 0.94,1.00; p = 0.05). Odds of reporting paternity leave were also lower if the baby had been born longer ago (AOR 0.93 for each month's increase in age; 95 % CI 0.89,0.96; p < 0.01); if the birth was in summer (AOR 0.65 compared to winter births; 95 % CI 0.44,0.97; p = 0.03); in fathers with lower education (AOR 0.56 for GCSE-level (age 16) education compared to a degree; 95 % CI 0.37,0.87; p < 0.01) and in fathers born outside the UK (AOR 0.49; 95 % CI 0.27,0.90; p = 0.02).

### 3.3 Association between paternity leave and measures of mental wellbeing and mental health

The sample for the IPWRA analysis was balanced after weighting, with standardised differences of <0.1 for all covariates in father and mother samples. Overall, we found no strong evidence of association between reported paternity leave uptake and mental wellbeing in either fathers or mothers (Table 3). Mental wellbeing scores were slightly higher among fathers who took paternity leave but the difference was small and not statistically significant (0.75-point difference in SF-12 MCS; 95 % CI -0.24,1.75; p = 0.13). Results were similar for the secondary outcome: fathers who took paternity leave had a non-significantly lower probability of screening positive for mental illness under the GHQ-12 (risk difference (RD) −0.03; 95 % CI -0.08,0.01; p = 0.15; see appendix E3). Among mothers, there was very little evidence of any association between the father of their baby taking paternity leave and either their SF-12 MCS score (−0.21 points; 95 % CI -1.37,0.95; p = 0.73) or GHQ-12 caseness (RD 0.01; 95 % CI -0.04,0.06; p = 0.76; see appendix D3).

**Table 3:** Difference in mental wellbeing score (SF-12 MCS) associated with taking paternity leave for fathers, mothers and subgroups (method: IPWRA using unweighted data).

Population	Difference in fathers' SF-12 MCS score if they took paternity leave (comparator: no leave)					Difference in mothers' SF-12 MCS score if their partner took paternity leave (comparator: no leave)				
	Mean difference in score	Robust standard error	p	Lower limit (95 %)	Upper limit (95 %)	Mean difference in score	Robust standard error	p	Lower limit (95 %)	Upper limit (95 %)
All complete cases	0.754	0.507	0.137	−0.240	1.748	−0.206	0.592	0.728	−1.367	0.954
All cases (missing covariate data imputed by MICE)	0.756	0.467	0.106	−0.160	1.671	−0.298	0.547	0.585	−1.370	0.773
Above median income	1.435	0.604	0.018	0.251	2.619	0.055	0.813	0.946	−1.539	1.649
Below median income	−0.281	0.651	0.665	−1.556	0.994	−0.027	0.813	0.973	−1.620	1.566
UK born	0.580	0.566	0.306	−0.530	1.689	0.075	0.694	0.914	−1.285	1.435
Not UK born	1.064	0.990	0.283	−0.877	3.005	0.436	1.162	0.707	−1.842	2.715
First-time parent	0.827	0.905	0.361	−0.947	2.602	0.409	1.147	0.722	−1.840	2.658
Not first-time parent	0.503	0.529	0.341	−0.533	1.540	−0.215	0.676	0.750	−1.540	1.109
≤2 weeks paternity leave	0.779	0.529	0.141	−0.258	1.816	−0.468	0.616	0.448	−1.674	0.739
>2 weeks paternity leave	0.888	0.627	0.157	−0.341	2.117	0.804	0.706	0.255	−0.580	2.188

### 3.4 Subgroup analyses

We found strong evidence of an association between paternity leave and better mental wellbeing (1.43-point difference in SF-12 MCS; 95 % CI 0.25,2.61; p = 0.02) in fathers with above-median household incomes. For fathers with below-median incomes, taking paternity leave was associated with lower mental wellbeing, but the strength of evidence of this association was very weak (−0.28 points; 95 % CI -2.49,0.65; p = 0.25). For mothers, there was no strong evidence of association between their partner's paternity leave and their mental wellbeing in either income group.

Subgroup analysis did not find any statistically significant association between paternity leave and mental wellbeing for parents born outside the UK; for first-time parents; or for families taking different lengths of paternity leave.

### 3.5 Sensitivity analyses

Results were robust to a wide range of sensitivity analyses, with similar point estimates and overlapping confidence intervals for the association between paternity leave and the primary outcome (SF-12 MCS) among both fathers and mothers. This was also the case for the secondary outcome (GHQ-12 caseness), and for both the weighted and the unweighted datasets, where models could be identified. The inclusion of people with imputed covariate data also resulted in similar findings.

Using weighted data in subgroup analysis led to a finding of better mental wellbeing among fathers who had taken >2 weeks of leave (1.9 points; 95 %CI 0.35,3.48; p = 0.02; see appendix table E1) and in mothers whose partners had taken >2 weeks (1.7 points; 95 % CI -0.002,3.34; p = 0.05). The latter finding was not statistically significant at the 95 % level.

**Fig. 1:**
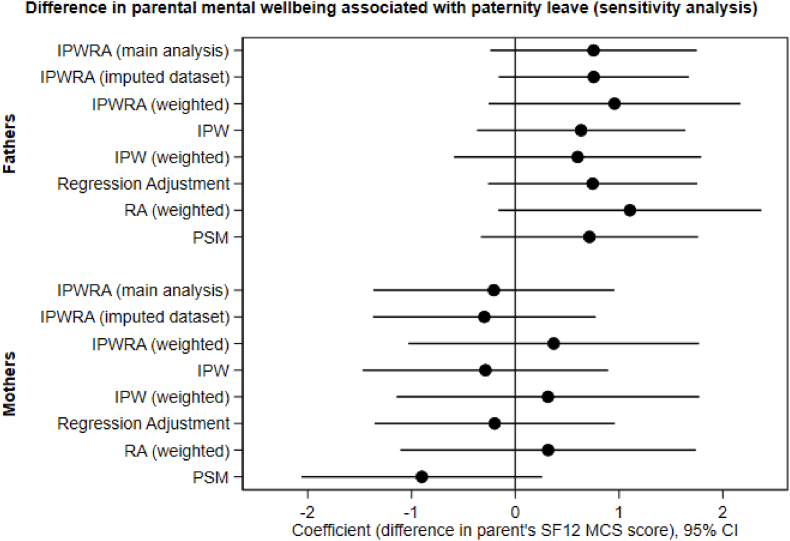
Coefficient plot showing results from different estimators of the association between paternity leave and mental wellbeing (sensitivity analysis).

Fig. 1 shows the range of estimates from different models used in sensitivity analyses for the primary outcome. Results from all models for both primary and secondary outcomes for fathers, mothers, and subgroups are in appendices E1-E4.

## 4 Discussion

We did not find strong evidence of association between paternity leave uptake and mental wellbeing or mental health of new fathers or mothers. Our findings suggest that the short (≤2-week) periods of paternity leave taken by most fathers in this sample did not have a large effect on these outcomes.

For mothers, our finding of no association in any subgroup means that the overall evidence remains unclear. Some previous research has found take-up of paternity leave to be associated with higher odds of depression for mothers (Barry et al., 2023). The authors have suggested that this might be due to higher uptake of paternity leave among fathers whose partners already had poor mental health. We adjusted for this by including mothers’ prior mental and physical wellbeing in our models, but still found no evidence of better mental health among those whose partners took paternity leave.

Theory suggests that paternity leave might improve maternal mental health - in part - by increasing paternal involvement in childcare. Protected leave time for fathers increases fathers’ relative contributions to childcare, and those who take longer leave tend to be more involved (Schober & Büchau, 2022). In our sample, the leave taken by most fathers may have been too short to change childcare arrangements. Our sensitivity analysis using weighted data gave some suggestive evidence of association between longer paternity leave and better mental health and this warrants further investigation.

For fathers, although we found no association overall, subgroup analysis highlighted an important inequality. For those in higher income households, mental wellbeing scores were significantly better with paternity leave than without it. This could be due to the relatively low rate of paternity pay available under UK policy. Decreases in income are associated with worsening mental health (Thomson et al., 2022), which may offset any benefits arising from paternity leave in lower-income households. Those with higher incomes may be better able to maintain good living standards during and after paternity leave. It is also possible that they also have other advantages which may be protective of mental health and which we were unable to control for, such as more favourable employer leave policies and access to childcare.

Overall, we examine a very limited form of paternity leave, and we find very limited evidence of association with either mental health or wellbeing outcomes. This could be analogous to the evidence about child and maternal health effects from maternity leave (or generic parental leave, which in practice is usually taken by mothers). To make the most difference to health and wellbeing outcomes, parental leave should be adequately paid and of sufficient duration: a recent systematic review concluded that maternal mental health benefits of parental leave are clearer if it is paid and if it lasts for at least 2–3 months (Heshmati et al., 2023). Similarly, unpaid parental leave seems to have few benefits for infant health and may widen inequalities (Nandi et al., 2018; Rossin, 2011). Further quantitative research on longer and better-paid paternity leave would complement this evidence on maternity and parental leave. Recent qualitative research found fathers flourishing during extended leave (Hobbs, 2024). However, long leave is atypical and its possible effects across the population are under-explored.

We also found demographic, social and economic inequalities in uptake of paternity leave. Lower take-up of this benefit among those born outside the UK and those with lower levels of education is concerning, and reinforces existing UK and international evidence that there are inequalities in access to paternity leave (Koslowski & Kadar-Satat, 2019; Månsdotter et al., 2010) as well as in its possible effects.

Our study has several strengths. It uses validated measures of the wellbeing and mental health of both mothers and fathers. It examines paternity leave, an under-researched area. We could control for many potential confounders using data gathered before the child's birth and the sample was balanced on covariates after weighting. Findings were robust to many sensitivity analyses.

Nevertheless, it also has important limitations. Although the models adjusted for many variables, we used observational data and there could be unobserved confounding. Point estimates are consistently small, while confidence intervals are wide, so real differences could have been too small to detect in this sample. It is also possible that differences in mental wellbeing arose from paternity leave during a crucial time shortly after the birth, but were not sustained until the time of interview, on average more than six months later. Moreover, findings cannot be generalised to a UK population - even in the weighted sensitivity analysis - because of the necessary exclusion of fathers in unstable employment.

Finally, there are measurement limitations. There were differences in fathers’ reported uptake of paternity leave depending on the age of their baby and the season of birth. This could be due to recall bias; or because some fathers took paid holiday instead of paternity leave, which would have been unobserved in this study. Underreporting of time off for these reasons could have biased the study towards the null hypothesis. The outcome measures also have limitations; they are short quantitative questionnaires and cannot capture all dimensions of mental health and wellbeing.

## 5 Conclusion

Although some forms of parental leave have health benefits, the evidence specifically about paternity leave remains unclear. In our UK-based study population, short durations of paternity leave under a low statutory pay entitlement did not make a significant difference to mental health and wellbeing, except for fathers in high-income households. Although paternity leave has the potential to confer mental wellbeing benefits, the lack of effect for mothers and for lower income fathers in our sample suggests that policymakers in the UK would need to improve the design of these entitlements to reduce mental health inequalities. Rigorous evaluation of the effects of longer leave periods and higher paternity pay in settings where they are - or become - available would help to inform these policy decisions.

## CRediT authorship contribution statement

**Emily Humphreys:** Writing – original draft, Project administration, Methodology, Investigation, Funding acquisition, Formal analysis, Data curation, Conceptualization. **Stephen O'Neill:** Writing – review & editing, Supervision, Methodology, Conceptualization. **Veronique Filippi:** Writing – review & editing, Supervision, Project administration, Methodology, Funding acquisition, Conceptualization. **Emilie Courtin:** Writing – review & editing, Supervision, Project administration, Methodology, Funding acquisition, Conceptualization.

## Ethical statement

This project was approved by the London School of Hygiene & Tropical Medicine Research Ethics Committee (Ref: 26846-02).

## Declaration of competing interest

The authors declare that they have no known competing financial interests or personal relationships that could have appeared to influence the work reported in this paper.

## Data Availability

The authors do not have permission to share data.

## Footnotes

1 Legislative changes are under consideration in the Employment Rights Bill, 2024-25.

2 These numbers differ because people who did not complete the SF-12 questionnaire in the year after their child's birth were excluded; there were several households in which only one parent completed this survey instrument.
