# Supplementary material for: Paternity leave, mental health and wellbeing for new parents: evidence from a national survey in the UK
Source: SSM Popul Health. 2025 May 7;30:101811. doi: 10.1016/j.ssmph.2025.101811 (PMC12140065; doi:10.1016/j.ssmph.2025.101811)
Supplement: Multimedia component 1 [file mmc1.docx]

**Paternity leave, mental health and wellbeing for new parents: evidence from a national survey in the UK (appendices)**

**Appendix A: conceptual framework**


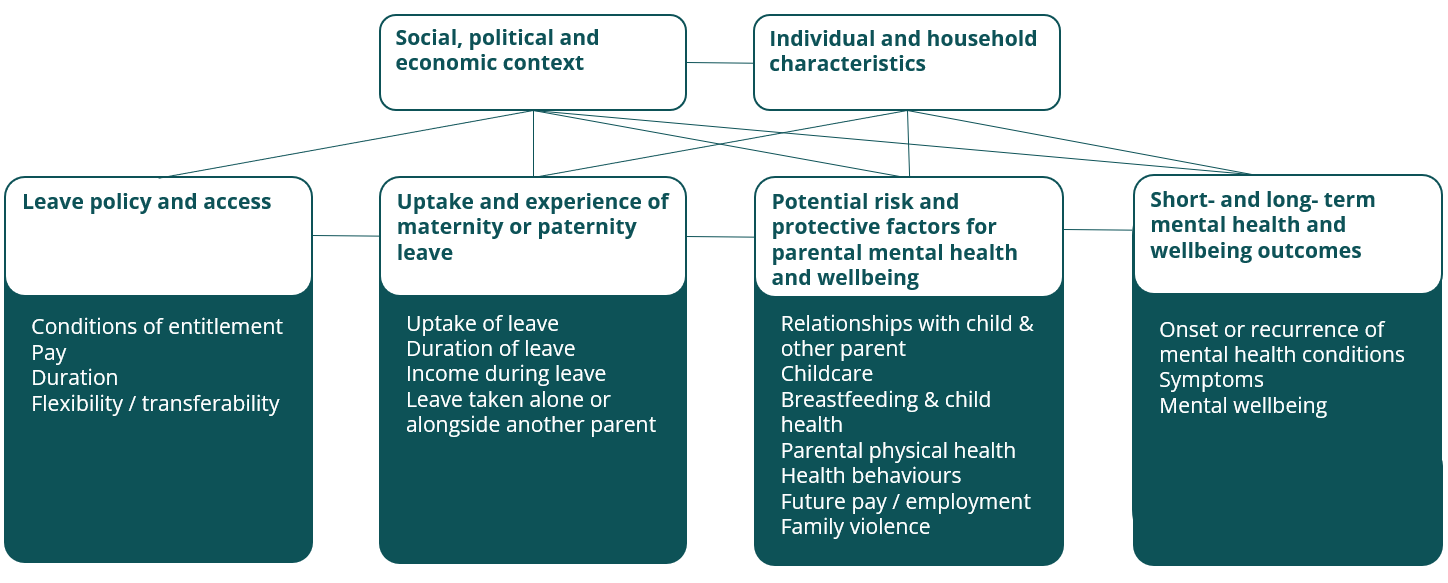


This framework adapts and builds on work by Andres and colleagues (2016)

**Appendix B: existing literature**

Table A1 describes existing research on this topic. Studies in the table were identified through:

1. inclusion in an existing systematic review (Heshmati et al, 2023), OR
2. structured database searches shown below in EconLit; MedLine; Embase; PsycINFO; Social Policy & Practice, Scopus or Web of Science, OR
3. they were referenced in a paper identified via 1) or 2)

Inclusion criteria were:

1. Quantitative, AND
2. Reports mental health or wellbeing outcomes for parents, AND
3. Compares groups with differences in access to or use of paternity or parental leave by fathers

*Table A1: summary of existing studies on paternity leave and mental health and wellbeing*

| **Lead author (year), journal** | **Country** | **Exposure / research question** | **Data source** | **N** | **Method** | **Population (outcomes)** | **Which parent gets leave?** | **Parent mental health outcomes** | **Parent mental health findings** | **Strengths** | **Limitations** |
| --- | --- | --- | --- | --- | --- | --- | --- | --- | --- | --- | --- |
| Barry (2023), Lancet Public Health | France | Exposure categories in this study are (1) has taken paternity leave (2) intends to take paternity leave (3) no paternity leave. | First wave of a cohort study of children born in 2011 (ELFE) | 10,975 | Imputed missing data Chi sq. & Kruskal-Wallis to compare exposure groups Logistic regression IPW | Mothers Fathers | Fathers | EPDS (binary outcome - depression)  2 months after child's birth | ***Fathers***  Lower odds of depression  **Mothers** Higher odds of depression | Lots of adjustment  Sensitivity tests Weighted  Balanced after adjustment Single timepoint | Cross-sectional (no prior mental health measure) Observational Unable to consider role of socioeconomic disadvantage |
| Bilgrami (2020), Health Economics | Australia | Two policy reforms.  **2011:** Paid Parental Leave (primary carer)  **2013:** Dad and partner pay (partner of eligible carer) | Household Income & Labour Dynamics in Australia (a national household-based panel survey). | 1,480 | Pre-post reform comparison  Lags of dependent variable to account for pre-natal and pre-pregnancy MH Sensitivity analysis including: proxy groups to check for time trends; exclusion of people who had births in more than one wave | Mothers | **PPL:** Primary carer **DAPP:** Partners of eligible carers | SF36 MCS Depression likelihood / severity at 4 categories (based on MHI score)  <12 months after birth | 14% decrease in likelihood of depression 2011 onwards; MCS score better but not significantly Introduction of DAPP leads to larger decrease in depression **Subgroups** Significant effects in first time mothers, not experienced mothers Significant effects in groups also entitled to employer-paid leave and job protection but not those with fewer existing entitlements | Many sensitivity analyses Sensitivity analysis with a comparator Considered different policy effects Models with and without covariates Use of lags | Not directly QE method - unable to get a comparator of ineligible Doesn't full disaggregate the two policy reforms |
| Bullinger (2019), Journal of Health Economics | USA | California (CA) Paid Family Leave policy reform (2004) <6 weeks of paid leave per parent.  Must have been paid $300 in base period No job protection Either 60% or 70% of weekly wage depending on earnings Capped at $1,216 per week | National Survey of Child Health (nationally representative sample in 2003, 2007, 2011-12) with 90,000 hh per wave. Supplemented by Behavioural Risk Factor Surveillance system to test for parallel trends | **Infants & mothers** 1,970 (CA vs neighbouring) 1,900 (CA vs other large states) 18,958 (CA vs all other states) **Fathers** 1,712 (CA vs neighbouring) 1,576 (CA vs other large) 16,255 (CA vs all) | Difference-in Differences (DiD) control group of people in other states Difference in DiD control group of older children and their parents | Children Mothers Fathers | Mothers Fathers | Self-reported very good mental health  Self-reported coping with day-to-day demands  At time child is aged 0-1 | Improvement in maternal mental health No effect on paternal mental health | Parallel trends from another dataset DD and DDD Several different control groups  Placebo tests using other states as the intervention group | Unvalidated measure of mental health |
| Cardenas (2021), Journal of Child and Family Studies | USA | Association between paid paternity leave of any duration on trajectories of perceived stress, fatigue and depression symptoms for mothers and fathers | 72 opposite-sex couples from within a longitudinal study of transition to parenthood, recruited through flyers in health settings, social media.  Prenatal and 6m lab visit + 12m follow-up survey | 72 | Longitudinal within-person approach Compares 'trajectories' rather than outcomes ANOVA / ANCOVA | First-time mothers First-time fathers | Fathers | Depressive symptoms  6m post birth and 12m post birth | **Fathers** - lower change in self-reported stress and fatigue in the leave group - no significant difference in depressive symptoms  **Mothers** - lower increase in stress and depressive symptoms in leave group - no significant difference in fatigue  Results mainly unchanged after controlling for length of leave | Explicit focus on paid leave  Also compares unpaid/any leave Longitudinal trajectories (pre-post birth) | Sample size  Unclear whether follow-up is 6m or 12m |
| Feldman (2004), Journal of Applied Developmental Psychology | USA | Correlates of duration of maternity and paternity leave and assessing factors related to more successful adaptation to reemployment in dual earner families. | Participants recruited from a birth record database in New Haven (1996-1998). Eligibility is first-time dual-earner families after the mother has returned to work, with child aged 3-5m. | 98 | Hierarchical regression. | First time mothers  First time fathers  Mother has resumed employment | Mothers Fathers | Beck Depression Inventory 3-5m post birth | **Mothers** Lower maternal depression associated with longer maternity leave **Fathers** No significant association between depression and length of paternity leave | Looks at both mothers and fathers  Considers duration of leave in days | Hard to follow  Babies aged 3-5 months; leave incomplete at that stage? |
| Honkaniemi (2022), J Epidemiol Community Health | Sweden | Policy reform for one month “daddy quota” (use-it-or-lose-it paternity leave) in 1995  Effects in Swedish-born and migrant populations | Linked registers: Total Population Register Multigenerational register Longitudinal Integration Database for Health Insurance and Labour Market Studies Medical Birth Register National Patient Register Cause of Death Register | 198,589 | Interrupted time series | First-time fathers | Fathers | Psychiatric hospital admissions over 3 years of follow-up | **Swedish-born** Increase in uptake from 63.6% to 86.4%, no difference in admissions **Migrant** Increase in uptake from 37.1% to 51.2% Stepwise decrease of 2.39 hospitalisations per 1000 person-years Slope decrease for every subsequent month of childbirth Some attenuation in sensitivity analyses | QE design  Large sample | Potential confounding by a move to care in the community and economic crisis  Results not holding in some robustness checks Also found a significant result on the pseudo-intervention date |
| Irish (2021), Am J Prev Med | USA | California Paid Family Leave (PFL) policy reform (2004) 6 weeks at 55% earnings for parents  New Jersey PFL (2009) 6 weeks at 2/3 earnings | National Health Interview Survey, an annual cross-sectional survey | 23,638 adults (parents of <2yo) 15,987 children | DiD Subgroups by parent sex, which intervention state, race/ethnicity, household income tertiles, marital status, parent age | Parents Children | Mothers Fathers | Kessler 6 (parents)  <2y after birth | Decreased psychological distress for both mothers and fathers White and middle-income parents experienced greater improvements | DiD  Subgroups  Two different states  Check for parallel trends Large sample | Don't know which parent was working Some of parallel trends didn't hold |
| Lee (2020), Soc Sci Med | USA | California PFL (2004) reform. <6 weeks of paid leave at 55% of weekly earnings for workers. Near-universal for private sector workers but state and local workers need to opt in. No job protection (though many are covered by FMLA). Range of outcomes. | Panel Study of Income Dynamics, a national longitudinal dataset. Used data from 1993 - 2017. | 6,690 | DiD; control group of people in states without a PFL policy. | Mothers Fathers | Mothers Fathers | Kessler-6 score  <2y after birth | **Overall** Increase in self-reported good/very good health Decreased psychological distress Decreased alcohol consumption  **Mothers** Only mothers had significant reduction in psychological distress  ***Fathers*** Fathers had greater reductions in obesity and alcohol | DiD method Large sample MICE sensitivity analysis  Some assumption tests ok  Range of outcomes | MH score only obtained for head of household Cross-over effects from both parents being eligible  Some differences in pre-post sample composition in age and race Placebo intervention dates did not all have null results  No data on uptake, only date |
| Lidbeck (2018), Journal of Reproductive and Infant Psychology | Sweden | Equal sharing of leave (40-60% each) vs non-equal sharing (mothers >60%). Based on planned division of leave. | Parents of babies at 25/83 child health centres in SW Sweden; cohabiting and fluent in Swedish. Recruited at 4m, questionnaire at 6m and 18m. Data collected Jan 2011-Jan 2013. | 280 complete cases | Multiple linear regression with change in parenting stress between 6m and 18m as dependent variable | Mothers  Fathers | Mothers  Fathers | SPSQ  6m & 18m after birth | **Fathers** Equal sharers are less affected by parenting stress **Mothers** No association | Both parents  Explicitly considers division of responsibility as outcome Validated stress questionnaire | Sample quite small; only 58% response rate. |
| Mansdotter (2010), Social Science & Medicine | Sweden | Association between 'masculinity' ages 18-19; paternity leave in 1988-90; and mortality 1991-2008. | Masculinity data from military Conscription Register; paternity leave from Social Insurance Register; mortality from the National Cause of Death Register + confounders data on income from the Income and Assets Register and education/occupation from the Swedish National Population and Housing Census. | 72,569 | Crude and multivariable logistic regression on masculinity + all-cause and cause-specific mortality; then paternity leave categories (0 days; 1-30; 31-135; >135) compared with masculinity ranks; then logistic regression with mortality as outcome and paternity leave as independent variable, stratified by level of masculinity. | Fathers | Fathers | Suicide mortality  Alcohol mortality up 20y | Crude OR has lower all-cause mortality for fathers who took paternity leave. AOR non-significant association w/ lower all-cause mortality and most cause-specific mortality, but 1-10 days paternity leave associated with higher AOR of suicide.  Masculinity not important for future uptake of paternity leave and paternity leave doesn't mediate association between masculinity and mortality. | Large population | Masculinity could affect probability of parenthood  'Masculinity' measure is questionable. |
| Nishigori (2020), J Matern Fetal Neonatal Med | Japan | Prevalence and factors associated with paternal postpartum depression at 1 and 6 months (includes paternity leave yes/no). | Adjunct to a prospective birth cohort study, the Japan Environment and Children's Study (data collected Jan 2011-Mar 2014); adjunct study is with participants from Miyagi | 1,330 | Crude and multivariate logistic regression; many covariates. | Fathers | Fathers | Japanese EPDS, 6m after birth | No significant difference (lower odds of depression for those who did not take leave p=0.36) | Only study in Japan EPDS Many covariates, including maternal EPDS scores and prebirth K6 | No maternal outcomes  Leave not defined (not main RQ) |
| Perry-Jenkins (2017), Community, Work and Family | USA | Association between various flexible working policies (at workplace level) with mental health for both mothers and fathers, including crossover effects | Interviews and questionnaires embedded in a larger longitudinal study of working class couples in the transition to parenthood, collected 1999-2002. Exposure variable is continuous measure of leave duration (in weeks for mothers and days for fathers). | 125 couples | Multilevel linear modelling examining workplace policies as a predictor of depressive symptoms and anxiety at three time points from return to paid employment to 1 year postpartum | Working class mothers Working class fathers | Mothers Fathers | CES-D depression symptoms  Spielberger's State-Trait Anxiety Inventory  Three times: - 1 month after mother's return to employment - Baby aged 6 months - Baby aged 1 year | **Mothers** Depression: no association with maternity leave or paternity leave length Anxiety: No association with maternity leave or paternity leave length **Fathers** Depression: No association with mat leave or pat leave length Anxiety: longer maternity leave lowers fathers’ anxiety; no association with paternity leave` Results refer to both initial level (RTW) and rate of change in outcome | Variety of policies  Low income focus  Duration of leave as exposure Other findings that different flexible workplace policies improve depression and anxiety for mothers | Excluded mothers who didn't return to work by 6m No comparator with higher income  Small sample  Generalisability Old data |
| Philpott (2018), Midwifery | Ireland | Prevalence and risk factors for paternal postnatal depression | Simple random cross-sectional sample of fathers >18 whose children were born in the last year from a primary care centre in Ireland | 100 | Chi squared and logistic regression | Fathers | Fathers | EPDS (paternal postnatal depression - binary outcome), <1y after birth | Employed fathers receiving paternity leave had lower prevalence (4.2%) than those not receiving paternity leave 19.4%). | 77% response rate | Sample size  Observational Results not fully tabulated  Not very heterogeneous population Self-report  Questionnaire not validated |
| Redshaw (2013), BMC Pregnancy & Childbirth | UK | Demographic factors (association with paternal engagement) Paternal engagement  Paternity leave | Survey data collected in 2010 drawn from random sample of new mothers in 2009. | 5333 mothers (4616 fathers) | Chi sq. Logistic reg GLM | Mothers of babies aged 3m | Fathers | Feeling well Psychological symptoms Self-reported depression at 1m & 3m | Lower depression at 1 and 3 months in multiparous mothers whose partner took 2 weeks of leave.  Difference not significant in primiparous mothers or those with shorter/longer paternity leave. | Sample size  UK  Different leave durations | Low response rate (55%) No weighting Cross-sectional  Adjustment variables not clear  Unvalidated outcome measures |
| Seimyr (2004), J Psychosom Obstet Gynecol | Sweden | Investigating period and point prevalence of maternal depression at >wk30 of pregnancy and again at 2m and 1y postpartum. | Survey of all Swedish-speaking women at 6 antenatal clinics October 1993-March 1994 | 434 gave consent; response rate 69% -75% across three questionnaires. | Longitudinal survey x 3 timepoints; surveying women and (optionally) their partners. | Mothers Fathers | Fathers | EPDS  2m pre-birth 2m post-birth 1y post-birth | Lower uptake of paternity leave among partners of depressed women.  Lower uptake of paternity leave among depressed men. | Pre- and post-birth data | Sample size Chi squared- no regression  Confusing presentation of results Very old data (and paternity leave uptake low) |
| Sejourne (2012), Journal of Reproductive and Infant Psychology | France | Association of perceived social support, paternity leave, and paternal participation in infant care with maternal PPD. | New survey data collected Nov2010-Mar2011 via three maternity hospitals in south of France. 350 approached (response 53%). Data at 2-5 days postpartum and 2 months postpartum. | 119 couples. | Multiple regression on mother's depression symptoms and logistic regression on binary indicator | Mothers | Fathers | EPDS score  EPDS (binary outcome - depression)  2m after birth | No association between paternity leave and depression score. Higher odds of depression if father had taken paternity leave. Perceived paternal involvement in infant care is protective against depression and associated with lower depression score. | Validated measures | Perceived paternal involvement a function of paternity leave Small sample size, poor response rate |
| Honkaniemi (2024), JAMA | Sweden | Effects of simultaneous parental leave use (any vs none; duration; and timing) | Medical Birth Register National Patient Register Swedish Social Insurance Microdata Registry  Longitudinal Integrated Database for Health Insurance and Labour Market Studies , for births in 2014 | 207,283 parental dyads | Logistic regression Sensitivity analysis using PSM  Stratified by parent Subgroup analysis by length and timing | Mothers Fathers | Both simultaneously | Outpatient visits for mental and behavioural disorders Prescriptions for antidepressants and anxiolytics  <1y after birth | **Mothers** Simultaneous leave use associated with higher odds of outpatient visits and psychotropic prescriptions.  Fully attenuated after controlling for previous mental health care use **Fathers**  No difference in mental healthcare use Higher odds of substance use and mood/affective disorder visits & of antidepressant prescribing after controlling for characteristics Substance misuse visits still significant association after controlling for previous mental healthcare use; others attenuated **Subgroup** Mums with <15 days simultaneous leave have higher antidepressant prescribing; 15-30 days, more outpatient visits for SUD. Dads with <15 days have decreased MH care use; 16-30 have increased use. | Sample size  Linked data PSM sensitivity analysis Able to control for previous mental healthcare use | Unmeasured confounding  Most significant associations attenuated after controlling for previous mental healthcare use  Unclear whether 'simultaneous leave' is substantially different from paternity leave (in practice) Results hard to interpret: are these actually increases in access to needed care as the authors say, or are they increases in need? |

Search terms

OVID databases: EconLit; MedLine; Embase; PsycINFO; Social Policy & Practice

| Set | Search Statement |
| --- | --- |
| 1. | maternity leave.mp. [mp=ti, ab, hw, tc, id, ot, tm, mf, ct, tn, dm, dv, kf, fx, dq, bt, nm, ox, px, rx, ui, sy, ux, mx, pt, an] |
| 2. | paternity leave.mp. [mp=ti, ab, hw, tc, id, ot, tm, mf, ct, tn, dm, dv, kf, fx, dq, bt, nm, ox, px, rx, ui, sy, ux, mx, pt, an] |
| 3. | parental leave.mp. [mp=ti, ab, hw, tc, id, ot, tm, mf, ct, tn, dm, dv, kf, fx, dq, bt, nm, ox, px, rx, ui, sy, ux, mx, pt, an] |
| 4. | maternity pay.mp. [mp=ti, ab, hw, tc, id, ot, tm, mf, ct, tn, dm, dv, kf, fx, dq, bt, nm, ox, px, rx, ui, sy, ux, mx, pt, an] |
| 5. | paternity pay.mp. [mp=ti, ab, hw, tc, id, ot, tm, mf, ct, tn, dm, dv, kf, fx, dq, bt, nm, ox, px, rx, ui, sy, ux, mx, pt, an] |
| 6. | childcare leave.mp. |
| 7. | family leave.mp. |
| 8. | 1 or 2 or 3 or 4 or 5 or 6 or 7 |
| 9. | matern*.mp. [mp=ti, ab, hw, tc, id, ot, tm, mf, ct, tn, dm, dv, kf, fx, dq, bt, nm, ox, px, rx, ui, sy, ux, mx, pt, an] |
| 10. | patern*.mp. [mp=ti, ab, hw, tc, id, ot, tm, mf, ct, tn, dm, dv, kf, fx, dq, bt, nm, ox, px, rx, ui, sy, ux, mx, pt, an] |
| 11. | mother*.mp. [mp=ti, ab, hw, tc, id, ot, tm, mf, ct, tn, dm, dv, kf, fx, dq, bt, nm, ox, px, rx, ui, sy, ux, mx, pt, an] |
| 12. | parent*.mp. [mp=ti, ab, hw, tc, id, ot, tm, mf, ct, tn, dm, dv, kf, fx, dq, bt, nm, ox, px, rx, ui, sy, ux, mx, pt, an] |
| 13. | father*.mp. [mp=ti, ab, hw, tc, id, ot, tm, mf, ct, tn, dm, dv, kf, fx, dq, bt, nm, ox, px, rx, ui, sy, ux, mx, pt, an] |
| 14. | 9 or 10 or 11 or 12 or 13 |
| 15. | mental health.mp. [mp=ti, ab, hw, tc, id, ot, tm, mf, ct, tn, dm, dv, kf, fx, dq, bt, nm, ox, px, rx, ui, sy, ux, mx, pt, an] |
| 16. | depression.mp. [mp=ti, ab, hw, tc, id, ot, tm, mf, ct, tn, dm, dv, kf, fx, dq, bt, nm, ox, px, rx, ui, sy, ux, mx, pt, an] |
| 17. | anxiety.mp. [mp=ti, ab, hw, tc, id, ot, tm, mf, ct, tn, dm, dv, kf, fx, dq, bt, nm, ox, px, rx, ui, sy, ux, mx, pt, an] |
| 18. | mental illness.mp. [mp=ti, ab, hw, tc, id, ot, tm, mf, ct, tn, dm, dv, kf, fx, dq, bt, nm, ox, px, rx, ui, sy, ux, mx, pt, an] |
| 19. | wellbeing.mp. [mp=ti, ab, hw, tc, id, ot, tm, mf, ct, tn, dm, dv, kf, fx, dq, bt, nm, ox, px, rx, ui, sy, ux, mx, pt, an] |

| 20. | well-being.mp. |
| --- | --- |
| 21. | self-harm.mp. [mp=ti, ab, hw, tc, id, ot, tm, mf, ct, tn, dm, dv, kf, fx, dq, bt, nm, ox, px, rx, ui, sy, ux, mx, pt, an] |
| 22. | self harm.mp. [mp=ti, ab, hw, tc, id, ot, tm, mf, ct, tn, dm, dv, kf, fx, dq, bt, nm, ox, px, rx, ui, sy, ux, mx, pt, an] |
| 23. | 15 or 16 or 17 or 18 or 19 or 20 or 21 or 22 |
| 24. | 8 and 14 and 23 |

Web of Science

((ALL=(mother* OR father* OR parent*)) AND ALL=(“mental health” OR “mental illness” OR depression OR anxiety OR “self-harm” OR wellbeing OR “well-being”)) AND ALL=(“maternity leave” OR “paternity leave” OR “parental leave” OR “childcare leave” OR “family leave”)

Scopus

( TITLE-ABS-KEY ( mother* OR matern* OR father* OR patern* OR parent* ) AND TITLE-ABS-KEY ( "mental health" OR "mental illness" OR "well-being" OR anxiety OR depression OR "self harm" ) AND TITLE-ABS-KEY ( "maternity leave" OR "paternity leave" OR "parental leave" OR "childcare leave" OR "family leave" ) )

**Appendix C: Sample construction**


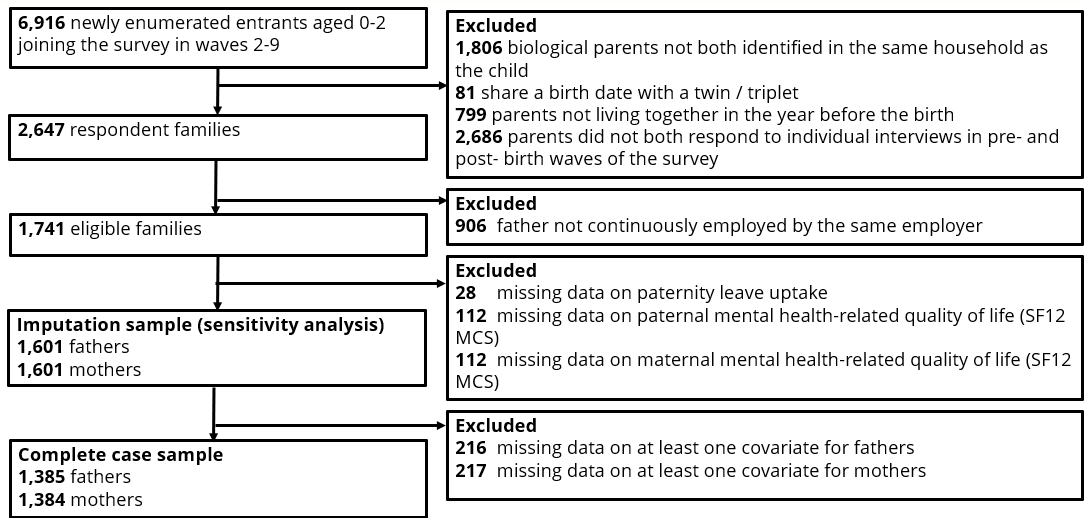


Appendix D: methodological details on regression adjustment

Regression adjustment addresses observed confounding by incorporating covariates in models to estimate potential outcome means for the whole sample with and without the exposure to paternity leave and calculating the difference between them; in IPWRA these models are augmented using inverse probability weights based on propensity scores for the exposure (StataCorp 2023, 392–406; Wooldridge 2007). IPWRA is doubly robust in that it generates consistent estimators if either the outcome model or the treatment model is incorrectly specified (Farrell 2015), for example by not having the right functional form.

Following Hirano and Imbens (2001), the propensity score is the probability of exposure to paternity leave (*T*) conditional on covariates (*X*):

$$ⅇ\left( x \right)=Pr\left( T=\left. 1 \right|X=x \right)$$

Propensity scores are in turn used to generate estimated inverse probability weights ($\hat{\omega})$ for each individual (*i*) in the sample:

$$\hat{\omega}\left( t,x \right)= \frac{t}{\hat{e}\left( x \right)}+ \frac{1-t}{1-\hat{e}\left( x \right)}$$

Hirano and Imbens estimate the following model, weighting by $\hat{\omega}$:

$$Y_{i}=\alpha_{0}+\tau\cdot T+\alpha_{1}^{'}X+\alpha_{2}^{'}\left( X_{i}-\bar{X} \right)\cdot T_{i}+ \varepsilon_{i}$$

Although this method originates in the causal inference field, causal interpretation would require assuming that selection into treatment is based on observable variables. This study is based on observational data. Not all relevant variables may be observed, so the approach is used here to increase the robustness of our associational estimates.

Appendix E: full outcome model results tables

*Table E1: association between paternity leave and parents’ mental wellbeing (SF12 score) by subgroup (method: IPWRA)*

|  |  | **Difference in fathers’ SF12 MCS score if they took paternity leave (comparator: no leave)** | | | | | **Difference in mothers’ SF12 MCS score if their partner took paternity leave (comparator: no leave)** | | | | |
| --- | --- | --- | --- | --- | --- | --- | --- | --- | --- | --- | --- |
| **Sample** | **Weighting** | **Co-efficient** | **Robust standard error** | **p** | **Lower limit (95%)** | **Upper limit (95%)** | **Co-efficient** | **Robust standard error** | **p** | **Lower limit (95%)** | **Upper limit** |
| All complete cases | Unweighted | 0.754 | 0.507 | 0.137 | -0.240 | 1.748 | -0.206 | 0.592 | 0.728 | -1.367 | 0.954 |
|  | Weighted | 0.957 | 0.619 | 0.122 | -0.257 | 2.170 | 0.370 | 0.715 | 0.605 | -1.031 | 1.770 |
| Multiple births excluded | Unweighted | 0.739 | 0.507 | 0.145 | -0.256 | 1.733 | -0.207 | 0.593 | 0.727 | -1.368 | 0.954 |
| **Above median income** | **Unweighted** | **1.435** | **0.604** | **0.018** | **0.251** | **2.619** | 0.055 | 0.813 | 0.946 | -1.539 | 1.649 |
|  | **Weighted** | **2.874** | **0.655** | **<0.001** | **1.590** | **4.159** | -0.053 | 0.879 | 0.952 | -1.776 | 1.671 |
| Below median income | Unweighted | -0.281 | 0.651 | 0.665 | -1.556 | 0.994 | -0.027 | 0.813 | 0.973 | -1.620 | 1.566 |
|  | Weighted | -0.920 | 0.801 | 0.251 | -2.491 | 0.651 | 1.397 | 1.075 | 0.194 | -0.709 | 3.504 |
| UK born | Unweighted | 0.580 | 0.566 | 0.306 | -0.530 | 1.689 | 0.075 | 0.694 | 0.914 | -1.285 | 1.435 |
|  | Weighted | 1.190 | 0.728 | 0.102 | -0.236 | 2.616 | 0.796 | 0.867 | 0.358 | -0.903 | 2.495 |
| Not UK born | Unweighted | 1.064 | 0.990 | 0.283 | -0.877 | 3.005 | 0.436 | 1.162 | 0.707 | -1.842 | 2.715 |
|  | Weighted | 1.136 | 1.900 | 0.550 | -2.588 | 4.859 | 1.664 | 1.829 | 0.363 | -1.920 | 5.248 |
| First-time parent | Unweighted | 0.827 | 0.905 | 0.361 | -0.947 | 2.602 | 0.409 | 1.147 | 0.722 | -1.840 | 2.658 |
|  | Weighted | 1.694 | 1.069 | 0.113 | -0.402 | 3.789 | Model not identified | | | | |
| Not first-time parent | Unweighted | 0.503 | 0.529 | 0.341 | -0.533 | 1.540 | -0.215 | 0.676 | 0.750 | -1.540 | 1.109 |
|  | Weighted | 0.363 | 0.687 | 0.597 | -0.984 | 1.710 | 0.147 | 0.821 | 0.858 | -1.462 | 1.756 |
| <=2 weeks paternity leave | Unweighted | 0.779 | 0.529 | 0.141 | -0.258 | 1.816 | -0.468 | 0.616 | 0.448 | -1.674 | 0.739 |
|  | Weighted | 1.269 | 0.663 | 0.056 | -0.031 | 2.569 | -0.307 | 0.754 | 0.683 | -1.784 | 1.170 |
| **> 2 weeks paternity leave** | Unweighted | 0.888 | 0.627 | 0.157 | -0.341 | 2.117 | 0.804 | 0.706 | 0.255 | -0.580 | 2.188 |
|  | **Weighted** | **1.912** | **0.800** | **0.017** | **0.345** | **3.479** | 1.670 | 0.853 | 0.050 | -0.002 | 3.341 |
| All cases (missing covariate data imputed) | Unweighted | 0.756 | 0.467 | 0.106 | -0.160 | 1.671 | -0.298 | 0.547 | 0.585 | -1.370 | 0.773 |

*Table E2: association between paternity leave and parents’ mental wellbeing (SF12 score) by modelling method (sample: all complete cases)*

|  |  | **Difference in fathers’ SF12 score if they took paternity leave (comparator: no leave)** | | | | | **Difference in mothers’ SF12 score if their partner took paternity leave (comparator: no leave)** | | | | |
| --- | --- | --- | --- | --- | --- | --- | --- | --- | --- | --- | --- |
| **Method** | **Weighting** | **Coefficient** | **Robust standard error** | **p** | **Lower limit (95%)** | **Upper limit (95%)** | **Coefficient** | **Robust standard error** | **p** | **Lower limit (95%)** | **Upper limit** |
| IPWRA | Unweighted | 0.754 | 0.507 | 0.137 | -0.240 | 1.748 | -0.206 | 0.592 | 0.728 | -1.367 | 0.954 |
|  | Weighted | 0.957 | 0.619 | 0.122 | -0.257 | 2.170 | 0.370 | 0.715 | 0.605 | -1.031 | 1.770 |
| IPW | Unweighted | 0.634 | 0.512 | 0.215 | -0.369 | 1.638 | -0.287 | 0.603 | 0.635 | -1.470 | 0.896 |
|  | Weighted | 0.601 | 0.608 | 0.322 | -0.590 | 1.792 | 0.314 | 0.744 | 0.673 | -1.144 | 1.772 |
| Regression adjustment | Unweighted | 0.746 | 0.514 | 0.147 | -0.262 | 1.753 | -0.198 | 0.591 | 0.737 | -1.356 | 0.960 |
|  | Weighted | 1.105 | 0.647 | 0.088 | -0.163 | 2.372 | 0.316 | 0.726 | 0.663 | -1.107 | 1.739 |
| Propensity score matching | Unweighted | 0.715 | 0.534 | 0.181 | -0.331 | 1.761 | -0.901 | 0.591 | 0.128 | -2.060 | 0.258 |

*Table E3: association between paternity leave and parents’ mental health (GHQ-12 caseness) by subgroup (method: IPWRA)*

|  |  | **Risk difference for probable mental illness (GHQ12 caseness) in fathers if they took paternity leave (comparator: no leave)** | | | | | **Risk difference for probable mental illness (GHQ12 caseness) in mothers if their partner took paternity leave (comparator: no leave)** | | | | |
| --- | --- | --- | --- | --- | --- | --- | --- | --- | --- | --- | --- |
| **Sample** | **Weighting** | **Coefficient** | **Robust standard error** | **p** | **Lower limit (95%)** | **Upper limit (95%)** | **Coefficient** | **Robust standard error** | **p** | **Lower limit (95%)** | **Upper limit** |
| All complete cases | Unweighted | -0.033 | 0.023 | 0.149 | -0.079 | 0.012 | 0.008 | 0.026 | 0.762 | -0.043 | 0.058 |
|  | Weighted | model not identified (perfect predictors in the outcome model) | | | | | -0.048 | 0.033 | 0.145 | -0.112 | 0.017 |
| Multiple births excluded | Unweighted | -0.033 | 0.023 | 0.154 | -0.079 | 0.012 | 0.009 | 0.026 | 0.727 | -0.042 | 0.060 |
| Above median income | Unweighted | model not identified (perfect predictors in the outcome model) | | | | | model not identified (perfect predictors in the outcome model) | | | | |
|  | Weighted | -0.024 | 0.037 | 0.504 | -0.096 | 0.047 | model not identified (perfect predictors in the outcome model) | | | | |
| Below median income | Unweighted | -0.012 | 0.024 | 0.625 | -0.059 | 0.035 | model not identified (perfect predictors in the outcome model) | | | | |
|  | Weighted | model not identified (perfect predictors in the outcome model) | | | | | model not identified (perfect predictors in the outcome model) | | | | |
| UK born | Unweighted | -0.036 | 0.026 | 0.164 | -0.086 | 0.015 | 0.009 | 0.030 | 0.751 | -0.049 | 0.068 |
|  | Weighted | model not identified (perfect predictors in the outcome model) | | | | | -0.045 | 0.039 | 0.256 | -0.122 | 0.032 |
| Not UK born | Unweighted | model not identified (perfect predictors in the outcome model) | | | | | model not identified (perfect predictors in the outcome model) | | | | |
|  | Weighted | model not identified (perfect predictors in the outcome model) | | | | | model not identified (perfect predictors in the outcome model) | | | | |
| First time parent | Unweighted | model not identified (perfect predictors in the outcome model) | | | | | model not identified (perfect predictors in the outcome model) | | | | |
|  | Weighted | model not identified (perfect predictors in the outcome model) | | | | | model not identified (perfect predictors in the outcome model) | | | | |
| Not first-time parent | Unweighted | -0.018 | 0.028 | 0.510 | -0.073 | 0.036 | model not identified (perfect predictors in the outcome model) | | | | |
|  | Weighted | model not identified (perfect predictors in the outcome model) | | | | | model not identified (perfect predictors in the outcome model) | | | | |
| <2 weeks paternity leave | Unweighted | model not identified (perfect predictors in the outcome model) | | | | | model not identified (perfect predictors in the outcome model) | | | | |
|  | Weighted | model not identified (perfect predictors in the outcome model) | | | | | model not identified (perfect predictors in the outcome model) | | | | |
| > 2 weeks paternity leave | Unweighted | model not identified (perfect predictors in the outcome model) | | | | | model not identified (perfect predictors in the outcome model) | | | | |
|  | Weighted | model not identified (perfect predictors in the outcome model) | | | | | model not identified (perfect predictors in the outcome model) | | | | |

*Table E4: association between paternity leave and parents’ mental health (GHQ-12 caseness) by method (sample: all complete cases)*

|  |  | **Risk difference for probable mental illness (GHQ12 caseness) in fathers if they took paternity leave (comparator: no leave)** | | | | | **Risk difference for probable mental illness (GHQ12 caseness) in mothers if their partner took paternity leave (comparator: no leave)** | | | | |
| --- | --- | --- | --- | --- | --- | --- | --- | --- | --- | --- | --- |
| **Method** | **Weighting** | **Co-efficient** | **Robust standard error** | **p** | **Lower limit (95%)** | **Upper limit** | **Co-efficient** | **Robust standard error** | **p** | **Lower limit (95%)** | **Upper limit** |
| IPWRA | Unweighted | -0.033 | 0.023 | 0.149 | -0.079 | 0.012 | 0.008 | 0.026 | 0.762 | -0.043 | 0.058 |
|  | Weighted | model not identified (perfect predictors in outcome model) | | | | | -0.048 | 0.033 | 0.145 | -0.112 | 0.017 |
| IPWRA (GHQ-12 caseness threshold >3) | Unweighted | -0.028 | 0.025 | 0.267 | -0.078 | 0.022 | 0.028 | 0.027 | 0.309 | -0.026 | 0.082 |
| IPW | Unweighted | -0.030 | 0.025 | 0.224 | -0.080 | 0.019 | 0.009 | 0.027 | 0.747 | -0.045 | 0.063 |
|  | Weighted | -0.020 | 0.035 | 0.568 | -0.088 | 0.048 | -0.027 | 0.036 | 0.447 | -0.098 | 0.043 |
| Regression adjustment | Unweighted | -0.022 | 0.023 | 0.335 | -0.068 | 0.023 | 0.014 | 0.027 | 0.588 | -0.038 | 0.067 |
|  | Weighted | -0.018 | 0.031 | 0.556 | -0.079 | 0.042 | -0.024 | 0.036 | 0.497 | -0.094 | 0.046 |
| Propensity score matching | Unweighted | -0.043 | 0.026 | 0.107 | -0.094 | 0.009 | -0.004 | 0.033 | 0.912 | -0.068 | 0.060 |

**References (appendices)**

Andres, Ellie, Sarah Baird, Jeffrey Bart Bingenheimer, and Anne Rossier Markus. 2016. “Maternity Leave Access and Health: A Systematic Narrative Review and Conceptual Framework Development.” *Maternal and Child Health Journal* 20 (6): 1178–92. https://doi.org/10.1007/S10995-015-1905-9.

Farrell, Max H. 2015. “Robust Inference on Average Treatment Effects with Possibly More Covariates than Observations.” *Journal of Econometrics* 189 (1): 1–23. https://doi.org/10.1016/j.jeconom.2015.06.017.

Hirano, Keisuke, and Guido W. Imbens. 2001. “Estimation of Causal Effects Using Propensity Score Weighting: An Application to Data on Right Heart Catheterization.” *Health Services and Outcomes Research Methodology* 2 (3/4): 259–78. https://doi.org/10.1023/A:1020371312283.

StataCorp. 2023. “Stata Casual Inferences and Treatment-Effects Estimation Reference Manual Release 18.” College Station,Texas: Stata Press.

Wooldridge, Jeffrey M. 2007. “Inverse Probability Weighted Estimation for General Missing Data Problems.” *Journal of Econometrics* 141 (2): 1281–1301. https://doi.org/10.1016/j.jeconom.2007.02.002.
